# Pheno- and Genotyping of Three Novel Bacteriophage Genera That Target a Wheat Phyllosphere *Sphingomonas* Genus

**DOI:** 10.3390/microorganisms11071831

**Published:** 2023-07-18

**Authors:** Leise Riber, Alexander Byth Carstens, Peter Erdmann Dougherty, Chayan Roy, Katharina Willenbücher, Frank Hille, Charles M. A. P. Franz, Lars Hestbjerg Hansen

**Affiliations:** 1 Department of Plant and Environmental Sciences, University of Copenhagen, Thorvaldsensvej 40, DK-1871 Frederiksberg, Denmark; alexander.carstens@plen.ku.dk (A.B.C.); ped@plen.ku.dk (P.E.D.); cr@plen.ku.dk (C.R.); lhha@plen.ku.dk (L.H.H.); 2 Department of Microbiology and Biotechnology, Max Rubner-Institut, Hermann-Weigmann-Strasse 1, 24103 Kiel, Germany; katharina.willenbuecher@mri.bund.de (K.W.); frank.hille@mri.bund.de (F.H.); charles.franz@mri.bund.de (C.M.A.P.F.)

**Keywords:** lytic phages, *Sphingomonas* phages, wheat flag leaf, TEM imaging, narrow host range, burst size, latency period, intergenomic similarity, genomic synteny, non-prolate Siphovirus

## Abstract

Bacteriophages are viral agents that infect and replicate within bacterial cells. Despite the increasing importance of phage ecology, environmental phages—particularly those targeting phyllosphere-associated bacteria—remain underexplored, and current genomic databases lack high-quality phage genome sequences linked to specific environmentally important bacteria, such as the ubiquitous sphingomonads. Here, we isolated three novel phages from a Danish wastewater treatment facility. Notably, these phages are among the first discovered to target and regulate a *Sphingomonas* genus within the wheat phyllosphere microbiome. Two of the phages displayed a non-prolate Siphovirus morphotype and demonstrated a narrow host range when tested against additional *Sphingomonas* strains. Intergenomic studies revealed limited nucleotide sequence similarity within the isolated phage genomes and to publicly available metagenome data of their closest relatives. Particularly intriguing was the limited homology observed between the DNA polymerase encoding genes of the isolated phages and their closest relatives. Based on these findings, we propose three newly identified genera of viruses: *Longusvirus carli*, *Vexovirus birtae*, and *Molestusvirus kimi*, following the latest ICTV binomial nomenclature for virus species. These results contribute to our current understanding of phage genetic diversity in natural environments and hold promising implications for phage applications in phyllosphere microbiome manipulation strategies.

## 1. Introduction

To date, bacteriophages (commonly also known as “phages”) are recognized as the most abundant and diverse biological entities in the biosphere [1]. These prokaryotic viruses exist in all known habitats that support bacterial growth, including terrestrial and aquatic environments [2,3]. Viral communities play a significant ecological role. With the unique ability of phages to infect and replicate within their host, typically with a narrow host range targeting only specific bacteria, they become important players in modulating the diversity of natural bacterial communities, thus having a notable impact on bacterial ecology and population dynamics [4,5]. 

The distinct host specificity of bacterial viruses makes the practical application of phages as biocontrol and biomanipulation agents highly attractive. Food industrial and agricultural practices are known to benefit from the ability of phages to alter the composition of bacterial communities [4,6,7], which makes the discovery and characterization of novel phages highly relevant.

Bacterial viruses are highly prevalent in natural environments, leading to phages constituting one of the greatest genomic reservoirs on the planet [8]. Yet, isolates of some phages remain surprisingly underexplored. Even with recent advances in high-throughput DNA-sequencing techniques, current genomic databases lack high-quality phage genome sequences that can be linked to specific environmentally important bacterial species. 

Bacterial members of the genus *Sphingomonas*, belonging to the *Sphingomonadaceae* family, are commonly known as an ecologically important group of bacteria that are ubiquitously distributed in diverse aquatic and terrestrial environments, as well as soil [9,10]. Other sources of *Sphingomonas* include the rhizosphere as well as the surfaces of various plants on which sphingomonads have been detected in rather high cell densities [11,12,13]. 

Interestingly, recent studies have identified the vital role of some *Sphingomonas* species in promoting plant growth during various abiotic stress conditions, a role attributed to their potential to produce plant growth hormones [14,15,16,17]. 

Overall, species belonging to the *Sphingomonas* genus are known to possess multifaceted functions. Importantly, they possess an extensive metabolic versatility with a prominent ability to degrade and transform a wide variety of natural organic compounds as well as environmental contaminants, i.e., xenobiotically produced compounds, such as biphenyl, chlorinated phenols, polycyclic aromatic hydrocarbons (PAHs), and various types of herbicides and pesticides [18,19,20,21]. On that note, members of the *Sphingomonas* genus have gained recent attention as being strong candidates for a wide range of biotechnological applications, with current utilization in bioreactors for enzyme production and in situ bioremediation practices [22,23,24].

Despite bacterial members of the genus *Sphingomonas* being widely prevalent in multiple environmental habitats, the isolation and characterization of phages targeting species of this genus remain sparse. Currently, there is a paucity of sequenced genome data of phages targeting *Sphingomonas* species and only a few have been fully sequenced [25]. 

The isolation of *Sphingomonas*-infecting phages originating from aquatic environments has been described in two studies with the main focus on morphology and infection kinetics [26,27], whereas morphology, host range, and phylogenetic diversity were described for *Sphingomonas* phages isolated from floodwater of a Japanese paddy field [28]. Recently, an in-depth genomic and proteomic characterization of the phage Lacusarx, which infects a *Sphingobium* species of the *Sphingomonadaceae* family, was reported [29]. Finally, physiological and genomic characteristics of the lytic phage vB_StuS_MMDA13, shown to infect *Sphingomonas turrisvirgatae*, revealed a very low similarity to other known phage genomes [25]. 

In this study, we describe the isolation and molecular characterization of the three newly discovered lytic phages *Longusvirus carli*, *Vexovirus birtae*, and *Molestusvirus kimi*, which were isolated from a Danish wastewater treatment facility. All phages possess the ability to infect and replicate on a bacterium from the *Sphingomonas* genus originating from the wheat phyllosphere, chosen based on the current status of wheat being an increasingly important crop worldwide [30]. The wheat phyllosphere microbiome, of which sphingomonads are potential keystone species [31,32], is believed to play a crucial role in extending the plant phenotype [33], hence implicating plant productivity and health. Yet, little is known about the microbial components that target and regulate the wheat phyllosphere microbiome, such as phages. 

All three phage genomes were described bioinformatically, whereas morphological features were described for only two phages due to the instability and consequent loss of the phage *Vexovirus birtae*. In brief, intergenomic studies revealed very little nucleotide sequence similarity between the isolated phages, and also to publicly deposited phage genomes, suggesting that the isolated phages represent three novel genera of viruses. Both the *Longusvirus carli* and *Molestusvirus kimi* phages were found to display a non-prolate Siphovirus morphotype. Despite the physical loss of the phage *Vexovirus birtae*, we do believe that these results contribute to a growing body of knowledge on the taxonomic diversity and ecological role of phages in natural environments—in particular, of those that might assist in regulating keystone bacterial members of the wheat phyllosphere microbiome.

## 2. Materials and Methods

The following describes the isolation and genome sequencing of both the bacterial *Sphingomonas* sp. host and the phages targeting this. We proceeded with Transmission Electron Microscopy (TEM) examination of phage morphology, bacteriophage infection kinetics, and host range determinations of the isolated vira. Detailed information on the methodology follows in the respective subsections below.

### 2.1. Isolation, Growth Conditions, and Genome Sequencing of the Bacterial Sphingomonas *sp.* Host

The bacterial host, *Sphingomonas* sp. strain LR55, was isolated in June 2021 from the surface of flag leaves of the winter wheat cultivar Sheriff, grown in an experimental field in Hoeje Taastrup, near Copenhagen, Denmark (55°40′12.2″ N 12°18′13.0″ E). Wheat flag leaves were picked, pooled, and washed in 0.9% NaCl + 0.05% Tween80 prior to plating on *Sphingomonas* selective L9 agar minimal medium [34]. Incubation was performed aerobically at 20 °C for 48 h. Colonies were re-streaked for purification on the same medium and with the same conditions at least three times and then stored at −80 °C in 20% (*v*/*v*) glycerol. Further inoculation of the host strain for phage isolation, phage host range determination, morphological characterization, and infection kinetics was performed on R2A medium [35]. 

Prior to DNA sequencing, DNA was extracted from 2 mL of liquid culture of the host strain in R2A medium using the Genomic Mini AX Bacteria kit (A & A Biotechnology, Gdynia, Poland). DNA long-read sequencing was performed using a PromethION48 sequencer from Oxford Nanopore Technology (ONT) with PromethION R9.4.1 flow cell chemistry. For library building, the Rapid Barcoding Kit 96 V14 (SQK-RBK114.96) was used (Oxford Nanopore Technologies, Oxford, UK). Base calling was performed with Guppy (version 6.4.6). Draft genomes were assembled using Flye (version 2.9.2-b1786) [36], annotated with PROKKA (version 1.13.3) [37] and online with BlastKOALA [38]. Assembled and annotated genomes were submitted to the NCBI GenBank [39] under the Bioproject PRJNA976731. 

### 2.2. Isolation, Purification, and Sequencing of Phages

Samples for phage isolation were collected from the Danish wastewater (WW) treatment plant, Lynetten, near Copenhagen. To enrich for phages infecting the bacterial host, a modified version of a previously described phage enrichment protocol was used [40]. In brief, ten mL of WW sample was centrifuged at 5000× *g* for 15 min to remove particulates and added to 50 mL of liquid R2A medium [35] supplemented with 10 mM of CaCl_2_ and MgCl_2_ together with one mL of overnight culture of the bacterial *Sphingomonas* sp. host strain LR55. Following a 48 h incubation step at room temperature (approximately 22 °C), the culture was centrifuged at 5000× *g* for 15 min to remove bacterial debris and the supernatant, now containing enriched phage particles, was filtered using a 0.45 μm syringe PVDF filter (Merck Millipore, Darmstadt, Germany). A total 1 mL of overnight culture of the bacterial *Sphingomonas* sp. host strain LR55 (~10^9^ CFU/mL) was mixed with 100 μL of different dilutions of the filtered supernatant and plated using a slightly modified double agar overlay assay protocol [41] with R2A medium supplemented with 10 mM CaCl_2_ and MgCl_2_ and 0.4% agarose as top agar, and R2A medium as bottom agar. 

To ensure single phage isolates, five rounds of plating and picking individual phage plaques were conducted using the double agar overlay assay as described above [41]. 

Phage DNA was extracted using a method modified from previous work [42]. Briefly, 10 U of DNase I (Thermo Scientific, Waltham, MA, USA) was added to 400 μL of phage lysates (~10^10^ PFU/mL), followed by incubation at 37 °C for 1 h. DNase I was inactivated by addition of 40 μL of 50 mM EDTA (Thermo Scientific, Waltham, MA, USA). The phage solution was treated with 20 mg/mL Proteinase K (A & A Biotechnology, Gdynia, Poland) to a final concentration of 0.8 mg/mL and 10% SDS solution to a final concentration of 0.1%. The phage solution was incubated for 1 h at 55 °C to open phage capsids. The temperature was hereafter raised to 70 °C for 10 min to inactivate proteinase K. The DNA was purified and concentrated using a DNA Clean & ConcentratorTM-5 kit (Zymo Research, Irvine, CA, USA) according to the manufacturer’s protocol and eluted with 20 μL of PCR-grade water (Sigma-Aldrich, St. Louis, MO, USA). 

DNA sequencing libraries were prepared using the NEBNext Ultra II FS DNA Library Prep kit for Illumina (Illumina, San Diego, CA, USA) according to manufacturer’s instructions. Prepared libraries were sequenced in a 151 paired-end sequencing run using the Illumina iSeq platform (Illumina, San Diego, CA, USA). 

### 2.3. Transmission Electron Microscopy (TEM) Examination of Phage Morphology

For morphology analyses of the phage particles, transmission electron microscopy (TEM) imaging was performed. Phages *Longusvirus carli* and *Molestusvirus kimi*, stored at a high titer in SM buffer [43], were purified for TEM examination by caesium-chloride (CsCl) gradient ultracentrifugation in an Optima XE-90 ultracentrifuge (Beckman Coulter, CA, USA) at 28,000 rpm for 90 min at 10 °C using three density layers (1.3, 1.45, and 1.7 g/cm^3^) [44] and concentrated using Amicon Ultra-4 centrifugal cellulose units with a pore size of 50,000 NMWL (Merck Millipore, Burlington, MA, USA). The CsCl-purified phage samples were pipetted on 100-mesh copper grids coated with 17.5 nm carbon for absorption for 20 min. Subsequently, the carbon films were washed twice with deionized water and negatively stained for a few seconds with 2% uranyl acetate using a protocol modified from previous work [45]. Electron microscopy was performed on a Talos L120C transmission electron microscope (ThermoFisher Scientific, Eindhoven, The Netherlands) using a 4 k × 4 k Ceta camera (ThermoFisher Scientific, Eindhoven, The Netherlands) set to an acceleration voltage of 80 kV.

Phage head and tail sizes were analyzed using the TEM analysis software Velox v3.9.0 (ThermoFisher Scientific, Eindhoven, The Netherlands). The mean and standard deviation of at least 10 measured virions per phage sample were determined. The TEM pictures were manually improved using GIMP v2.10.32 for contrast increasing and brightness adjustment.

### 2.4. One-Step Growth Curve Experiments

One-step growth curve experiments were conducted using a previously described, but slightly modified, protocol [46] to determine the infection kinetics (i.e., burst size and latent period) of phages *Longusvirus carli* and *Molestusvirus kimi*. In brief, the bacterial host *Sphingomonas* sp. strain LR55 was grown in liquid R2A medium [35] in a shaking water bath at 25 °C, 120 rpm, to an OD_600_ of 0.24 (determined to correspond to 2.5 × 10^8^ CFU/mL) and infected at a multiplicity of infection (MOI) of 0.06 and 0.024 with phages *Longusvirus carli* and *Molestusvirus kimi*, respectively. The cultures were incubated with shaking at 120 rpm for 40 min at 25 °C to allow adsorption of phages to host cells. Following adsorption, 1 mL of each infected culture was centrifuged at 8000× *g* for 3 min to remove non-adsorbed phages. Pellets were resuspended in 1 mL of SM buffer [43], diluted by a factor E-04 in 30 mL of fresh liquid R2A medium [35], and incubated in triplicates in a water bath at 25 °C and at 120 rpm shaking. To determine the latency period and burst size for each phage, aliquots of 0.2 mL were removed over time, for a total of 10 h, and phage titer was determined in triplicate using the double agar overlay technique [41]. The latency periods were measured excluding the 40 min of phage adsorption. The average burst size for each phage (i.e., the average number of phages released per infected host cell) was computed as the ratio of the average phage titer after the rise period to the average phage titer during the latency period (directly representing the number of infecting phage particles due to removal of non-adsorbed phages prior to dilution) [47]. 

The adsorption efficacy of each phage was measured by counting unattached PFUs relative to total PFUs using the double agar overlay assay as described above. This was achieved by centrifugation of the samples at 12,000× *g* for 3 min at T = 0 min and T = 40 min and titering the PFUs of the supernatant (representing unattached phages) compared with the total PFUs without centrifugation (representing total phages). Measurements were performed in triplicates. 

### 2.5. Determination of Phage Host Range 

Host range determination was performed by spotting 20 μL of 100-fold dilutions (undiluted up to E-08 factor) of the phages *Longusvirus carli* and *Molestusvirus kimi* (~10^10^ PFU/mL) on lawns of different *Sphingomonas* wheat flag leaf isolates from our Laboratory collection as well as on lawns of 3 previously characterized *Sphingomonas* strains (Table 1; Section 3.5) using double agar overlay assays [41] with the same media and growth conditions as described in Section 2.2. The *Sphingomonas* wheat flag leaf isolates were obtained in June 2021 or 2022 from Danish experimental fields at various locations by a procedure similar to that of the *Sphingomonas* sp. host strain LR55 (see Section 2.1). Initial growth medium used for isolation included L9 minimal medium [34], R2A minimal medium [35], VL55 medium [48], and a plant extract medium consisting of a basal medium ((NH_4_)_2_HPO_4_ (0.05 g/L), CaCl_2_ (0.02 g/L), NaCl (0.1 g/L), MgSO_4_ (0.2 g/L), K_2_HPO_4_ (0.4 g/L), KH_2_PO_4_ (0.4 g/L), and Glucose (2.0 g/L), pH 7.2.) to which 30 g of crushed wheat leaves in 300 mL of water, filtered and autoclaved, was added. The genomes of the *Sphingomonas* wheat flag leaf isolates were nanopore sequenced using protocols similar to that of the *Sphingomonas* sp. host strain LR55 (see Section 2.1). Details on sampled locations are stated in Table 1; Section 3.5. 

Sensitivity to phage infection was assessed by observing either the formation of individual plaques or the presence of clear zones of phage lytic activity after 48 h of incubation at 20 °C of the spotted dilutions. When individual plaque formation was observed, the number of PFUs was titered and further subjected to relative efficiency of plating (EOP) analysis using triplicates. EOP was calculated as follows: average PFU on target bacteria/average PFU on host bacteria [49]. 

### 2.6. Genomic Analysis of the Isolated Phages

Clean Illumina reads of phage genomes were assembled using CLC Genomic Workbench V22 (QIAGEN, Aarhus, Denmark), as described previously [42]. Open reading frame (ORF) identification and annotation of assembled genomes was performed using RASTtk annotation server version 2.0 [50] with the modifications described elsewhere [42]. Assembled and annotated genomes of phages *Longusvirus carli*, *Vexovirus birtae*, and *Molestusvirus kimi* were assigned to GenBank with accession numbers OR225223, OR225224, and OR225222, respectively. 

Intergenomic similarities were calculated and visualized using the VIRIDIC web server [51] based on pairwise nucleotide comparisons between the five closest relatives to each phage identified using BLASTN [52]. BLASTP [53] was used to identify the five genes with the highest nucleotide sequence similarity to the DNA polymerase encoding genes as well as the large terminase genes of *Molestusvirus kimi* and *Vexovirus birtae* in the publicly available databases. To inspect the gene cluster conservation (or lack thereof), phages carrying these genes were compared using the clinker toolkit V0.0.27 [54].

## 3. Results and Discussion

### 3.1. Isolation, Sequencing, and Identification of the Bacterial Host, Sphingomonas *sp.* Strain LR55 

The bacterial host strain (LR55), a member of the *Sphingomonas* genus, was isolated on selective L9 media plates as orange-pigmented colonies from the surface of wheat flag leaves collected at a Danish experimental wheat field. Whole genome sequencing (WGS) using Oxford Nanopore Technology (ONT) yielded a total of 457 MB of data with an N50 value of 3,960,318 bp and a 111× sequencing coverage of the 4.07 Mbp genome, consisting of two contigs with a GC-content of 65.3%. Following taxonomical classification of the assembled genome using the GTDB-Tk database [55], the host strain, LR55, was classified as *Sphingomonas* sp. without being assigned to any known species level, hence representing a putatively novel *Sphingomonas* species not currently available in the genome taxonomy database. Prodigal gene prediction resulted in a total number of 4097 genes including 4024 protein coding sequence (CDS) regions and 73 non-coding RNAs (60 tRNAs, 12 rRNAs, and one tmRNA). No complete prophage genomes were detected in the host genome using PHASTER [56]. 

As detected by BlastKOALA (providing an annotation of 41.2% of the detected CDS), *Sphingomonas* sp. strain LR55 was found to harbor several catabolic genes potentially involved in the biodegradation and metabolism of xenobiotic compounds, as commonly seen in sphingomonads [23]. Multiple genes involved in the degradation of fluorinated and chlorinated organic compounds as well as aromatic hydrocarbons were found in the annotation; also, one gene that was possibly associated with the degradation of the persistent organic pollutant dioxin, a suspected cancer-causing agent [57], was detected. Interestingly, one strain of the *Sphingomonas* genus, *Sphingomonas wittichii* RW1, was previously described as a potent dioxin-degrading bacterium [58,59]. 

### 3.2. Identification of Phages Longusvirus carli, Vexovirus birtae, and Molestusvirus kimi and General Features of Phage Genomes 

Following a standard enrichment procedure for isolating phages from a Danish wastewater source, as described above using the wheat phyllosphere *Sphingomonas* sp. strain LR55 as host, several clearly visible plaques were obtained, of which 16 were chosen for further purification. A sequencing-based pre-screening revealed that two of the propagated phages belonged to the *Molestusvirus kimi* genotype, one belonged to the *Vexovirus birtae* genotype, whereas the rest belonged to the *Longusvirus carli* genotype. 

For an in-depth genomic analysis, sequencing of genomic DNA extracted from phages *Longusvirus carli*, *Vexovirus birtae*, and *Molestusvirus kimi* resulted in fully assembled genomes of sizes 57,382 bp; 45,617 bp; and 43,699 bp, respectively. The genomes of the three phages were all terminally redundant; however, no clear bias in the start position of reads could be found and, thus, the correct start/end possession of the genomes or any direct terminal repeats could not be identified. For *Vexovirus birtae* and *Molestusvirus kimi*, a putative small terminase was identified and chosen as the start site of the genomes. The genomic GC-content of the three phages ranged from 55.9% to 63.0% (*Longusvirus carli*, 63.0%; *Vexovirus birtae*, 57.2%; and *Molestusvirus kimi*, 55.9%), which is lower than the GC-content of the bacterial host (65.3%), as is often seen in phages [29,60,61]. 

**Figure 1:**
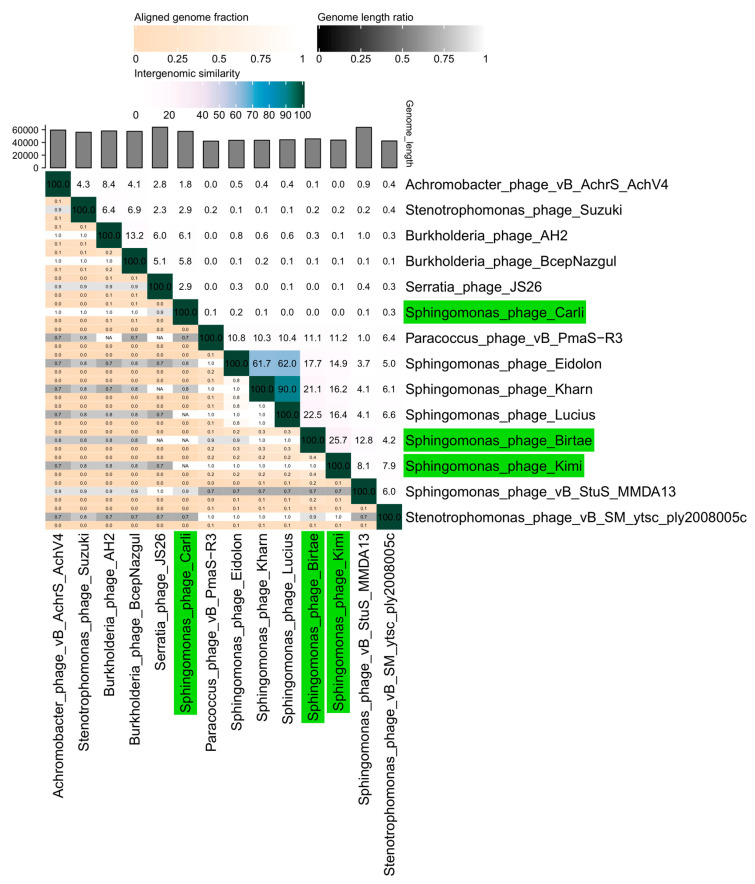
A VIRIDIC heat map [51] showing the intergenomic similarity of the isolated phages, *Longusvirus carli*, *Vexovirus birtae*, and *Molestusvirus kimi*, compared with the five closest relatives of each phage. Relevant pairwise nucleotide comparisons were identified and made using BLASTN [52]. Phages isolated in this study are colored green.

The phage *Longusvirus carli* shares very little nucleotide sequence similarity (less than 10%) to any known phage in publicly available databases and is the first representative of a completely new group of phages (Figure 1, BLASTN [52]). Only 7 of 87 open reading frames could be successfully annotated. The annotated genes are primarily involved in DNA replication and phage DNA packaging or represent putative structural genes. Because of the low sequence similarity to known phages and the lack of annotated genes, further genetic analysis was not attempted.

The phages *Vexovirus birtae* and *Molestusvirus kimi* are more closely related to each other (25% nucleotide similarity) than to any other known phage (Figure 1, BLASTN [52]). *Vexovirus birtae* and *Molestusvirus kimi* share some nucleotide sequence similarity (15–23%) to a group of other phages targeting bacterial species of the *Sphingomonas* genus (Eidolon, Accession number: MN734437.1; Kharn, Accession number: MN734439.1; and Lucius, Accession number: MN734438.1). However, both represent novel genera and possibly higher taxonomic classifications as well. 

**Figure 2:**
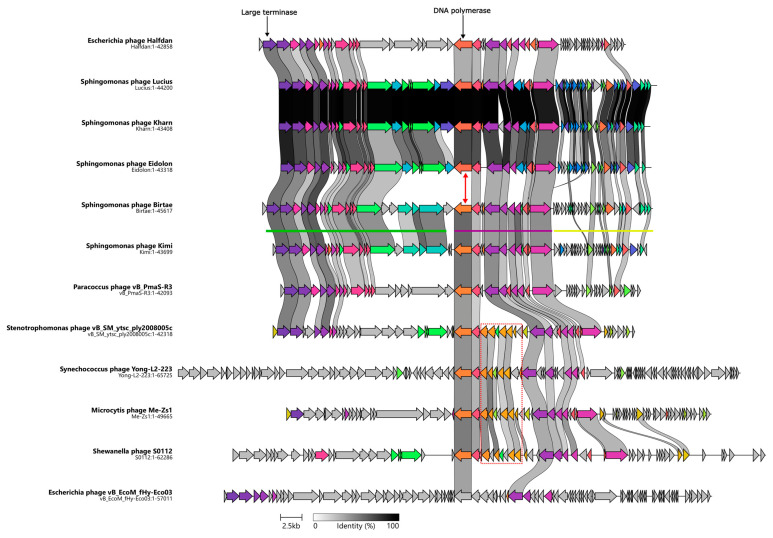
Clinker map showing the genomic synteny of the isolated phages, *Vexovirus birtae* and *Molestusvirus kimi*, and their closest relatives as well as the observed gene cluster conservation between these genomes. Genome sizes (bp) are listed below each phage. The DNA polymerase encoding gene of *Vexovirus birtae* and *Molestusvirus kimi* is distinct from that of the other *Sphingomonas* phages (red arrow). The red box highlights the queuosine biosynthesis operon. The green, purple, and yellow lines represent the different gene clusters found in the phages.

The genomes of *Vexovirus birtae* and *Molestusvirus kimi* can be divided into three gene clusters. The first cluster (Figure 2, green line) contains mostly structural genes as well as genes that are typically expressed late in the phage life cycle. Here, genes encoding the major capsid, small and large terminases, as well as various putative structural genes can be found. Most of the genes in this cluster are highly conserved between relatives but some of the genes towards the end of the operon are less conserved. The less-conserved genes contain some putative tail genes and might be involved in host recognition, which could partly explain their lack of conservation. 

The second cluster (Figure 2, purple line) contains a number of genes involved in DNA metabolism and DNA replication including the DNA polymerase, primase, and helicase encoding genes. Unlike the other two gene clusters, genes belonging to this cluster are mostly reversely orientated (i.e., encoded on the antisense strand). Most of the genes in this cluster are conserved with their closest relatives (Figure 2). However, a noticeable exception to this is the DNA polymerase encoding gene. The DNA polymerase encoding gene of *Vexovirus birtae* and *Molestusvirus kimi* does not share any homology to the DNA polymerase encoding genes of their closest relatives, the *Sphingomonas* phages Kharn (Accession: MN734439.1), Lucius (Accession: MN734438.1), and Eidolon (Accession: MN734437.1) (Figure 2, red arrow). Interestingly, notable homology is shared with the DNA polymerase encoding genes of their more distant relatives (Figure 2, BLASTP [53]). This might indicate a recent event of horizontal gene transfer of the DNA polymerase encoding gene. Interestingly, some of the close relatives contain a roughly 5 Kbp insertion of an entire queuosine biosynthesis operon into this gene cluster. This operon has previously been shown to be involved in DNA modification of phage DNA and protection against host restriction enzymes [62,63,64]. 

The final gene cluster (Figure 2, yellow line) consists primarily of small genes of unknown function. These genes are mostly poorly conserved and on average much smaller than genes in the other gene clusters. Yellow cluster genes have an average size of 342 bp compared with an average gene size of 963 bp for the rest of the genome. By comparing the stop codons applied in this gene cluster with those used by the gene clusters of the remaining genome, it becomes clear that there are some significant differences, e.g., in *Molestusvirus kimi*, 61% (19 of 31) of the ORFs in this particular gene cluster (Figure 2, yellow line) end with a TGA stop codon as compared with only 26% in the remaining genome. Even though this is a large difference, it is not evident that the difference in gene size is a result of mis-annotation caused by stop codon read-through. No tRNA genes or other signs of stop codon read-through were identified.

### 3.3. Phage Plaque and Virion Morphology Characteristics

**Figure 3:**
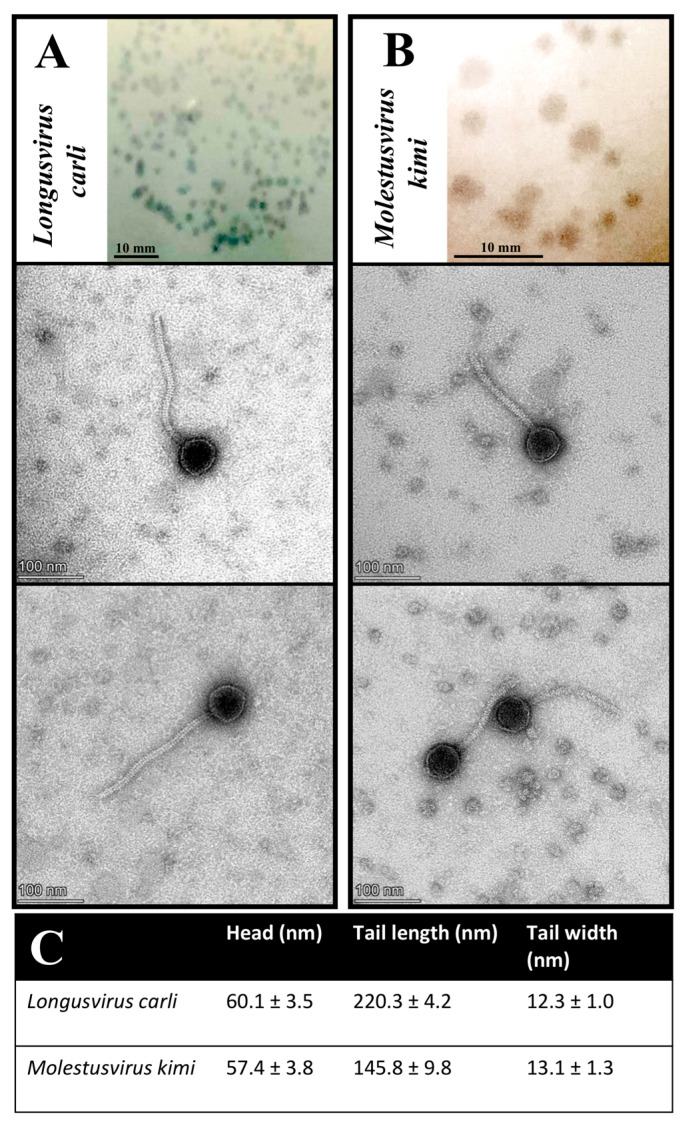
Plaque and virion morphology features of phages *Longusvirus carli* (**A**) and *Molestusvirus kimi* (**B**). (**Top Insert**) Phage plaque morphology on lawns of the bacterial host, *Sphingomonas* sp. strain LR55. Bars indicate 10 mm. (**Bottom Inserts**) Phage virion morphology as presented by transmission electron micrographs. Bars indicate 100 nm (**C**). Phage head and tail sizes analyzed using TEM analysis software. Presented are the mean and standard deviation of at least 10 measured virions per phage sample.

Following the initial isolation and DNA sequencing of *Longusvirus carli*, *Vexovirus birtae*, and *Molestusvirus kimi*, stability issues were unfortunately observed for *Vexovirus birtae*, which resulted in loss of infectivity of the phage lysate during storage, preventing further propagation and morphology analyses of this phage. However, prior to the physical loss of *Vexovirus birtae*, the phages *Longusvirus carli* and *Vexovirus birtae* were found to share a similar plaque morphology, both forming clear and circular plaques of around 0.8–2 mm in diameter on a bacterial lawn after incubation for two days at 20 °C (Figure 3A, top insert). In comparison, the phage *Molestusvirus kimi* formed larger, less clear, and circular plaques of around 1–4 mm in diameter on a bacterial lawn using the same incubation conditions (Figure 3B, top insert).

Transmission electron microscopy (TEM) imaging of at least 10 measured virions per phage sample revealed virion morphological features for the phages *Longusvirus carli* and *Molestusvirus kimi* typical of a non-prolate (B1) Siphovirus morphotype belonging to the class *Caudoviricetes*, with icosahedral heads and flexible, non-contractile tails of various lengths [65]. The phage *Molestusvirus kimi* has a short tail with a more rounded tip and is less pointy (Figure 3B, bottom inserts) compared with the phage *Longusvirus carli*, which displays a longer tail with a regular tail tip (Figure 3A, bottom inserts). For none of the phages were visible fibers detected (Figure 3A,B, bottom inserts; Figure 3C). 

Phages displaying a non-prolate (B1) Siphovirus morphotype are often isolated from various environmental habitats [66,67,68,69,70]. 

To our knowledge, however, the phages *Longusvirus carli* and *Molestusvirus kimi* are among the first characterized phages with a non-prolate Siphovirus morphotype that infect a member of the *Sphingomonas* genus. Recently, the phage vB-StuS_MMDA13, also targeting members of the *Sphingomonas* genus, was found to display a Siphovirus morphotype, yet with an icosahedral elongated prolate head [25]. Finally, another recent phage called Lacusarx, which targets members of the *Sphingobium* genus, was also found to display the head–tail geometry of the Siphovirus morphotype but with a notably elongated prolate capsid and more unusual morphology [29]. 

### 3.4. Phage Infection Kinetics

One-step growth curve experiments were conducted to determine the infection kinetics (i.e., burst size and latent period) of the phages *Longusvirus carli* and *Molestusvirus kimi*.

**Figure 4:**
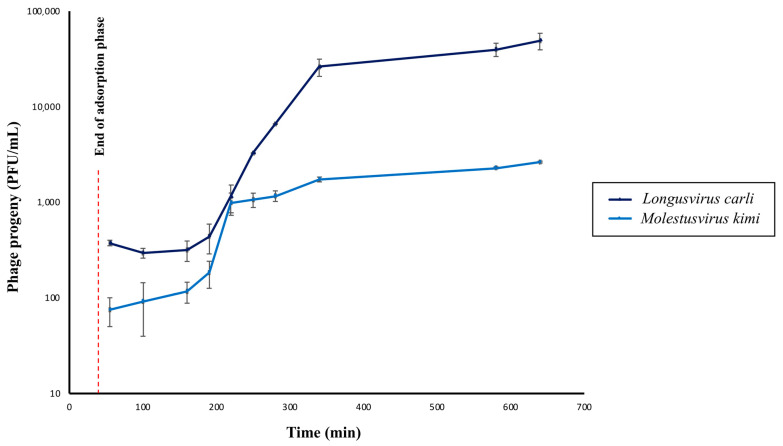
One-step growth curves of phages *Longusvirus carli* and *Molestusvirus kimi*. The concentration of total phage particles (i.e., phage progeny) at different timepoints following infection is shown. Data represent the average of three independent experiments. Vertical black bars indicate the standard deviation (SD) of each data point. Adsorption was initiated at Time = 0 min. The red dashed line represents the time point at which the adsorption phase ends (Time = 40 min).

Overall, both phages were shown to display latency periods of approximately 150 min, which is excluding the 40 min of adsorption prior to dilution and incubation of the phage–host infection mixtures. Interestingly, the latency period of the phage *Longusvirus carli* is followed by a long rise period of approximately the same duration as the latency period, whereas the rise period seems shorter for the phage *Molestusvirus kimi* (~30 min) (Figure 4). It can, however, be argued whether a slight increase in phage titer continues after 180 min for this phage, leading to a possibly extended rise period as well. These long latency and rise periods are not uncommon for slow-growing bacterial host strains (in this study, estimated generation time ~180 min), and previous studies have reported similar data for other *Siphoviridae* infecting, slow-growing *Alphaproteobacteria* [25,71,72]. 

The average burst size of the phage *Longusvirus carli* was determined to be approximately 117 virions per infected cell, whereas the average burst size of the phage *Molestusvirus kimi* was about 17 virions per infected cell. For both phages, computation of burst size included the last three and the first three sampling points for calculations of average phage titer after the rise period and during latency, respectively. It can be debated whether more or less sampling points should be considered for burst size calculations. Such changes, however, did not lead to any significant alterations in the range of reported burst sizes. 

Burst size estimates of prokaryotic cells in situ are highly variable and typically found in the range from a few to around 500 phages [73]. Generally, burst size is believed to be influenced by several factors, such as bacterial/viral size, metabolic activity of the host, as well as the characteristics of the phage and host [73]. Obviously, burst size differs between species; however, phages isolated from the same bacterial host and cultured in a similar nutrient-rich medium might also display different burst sizes [74]. 

For phages infecting bacterial members of either the *Sphingomonas* genus or the *Sphingobium* genus, burst sizes were previously estimated to an average of 30 and 46.5 virions per infected cell [25,29], respectively, which resembles similar orders of magnitude as seen in this study. 

Interestingly, the adsorption efficacy of the phages *Longusvirus carli* and *Molestusvirus kimi* was determined to be 62.9% ± 17.5 (SD) and 49.8% ± 16.3 (SD), respectively, following 40 min of incubation, which represents a slow rate of adsorption. Therefore, the rise period of *Molestusvirus kimi* likely represents the slow attachment rather than variation in eclipse periods between individual virocells. The long rise period of *Longusvirus carli* can only be partly explained by the slow attachment. 

Typically, phage adsorption rates are functions of several parameters, such as virion diffusion rates, host size and concentration as determining virion collision rates, phage affinity for attachment receptor sites, and likelihood of reversible attachment [75]. Hence, adsorption kinetics are expectedly highly variable, depending on the specific case of phage–host interaction. 

### 3.5. Determination of Phage Host Range

A total of sixteen strains, including thirteen recently sequenced *Sphingomonas* isolates obtained from the wheat phyllosphere from Danish wheat trial fields, and three *Sphingomonas* strains previously characterized as known degraders of chlorinated phenoxy herbicides (*Sphingomonas* sp. PM2 [76] and *Sphingomonas herbicidovorans* MH [77]) as well as dibenzofuran and dibenzo-*p*-dioxin (*Sphingomonas wittichii* RW1 [59]), were used to study the host range of the phages *Longusvirus carli* and *Molestusvirus kimi* (Table 1). Several wheat isolates obtained in a similar manner as the *Sphingomonas* sp. host (strain LR55) were chosen with the aim of detecting an increased phage susceptibility. The results, however, demonstrated that the lytic potential of the phage *Longusvirus carli* is exclusively restricted to the original *Sphingomonas* sp. host, whereas all other tested strains were insensitive to infection, suggesting a narrow host range of this phage. 

**Table 1:** Strains used for host range determination. “+” clear lysis zone; “-“ no lysis zone.

Strain Number/ Isolate	Sampled Location ^a^/ Year	Isolation Medium ^b^	GC-Content (%)	Genome Size (Mbp)	Accession Number ^c^ (GenBank)	Ref	Susceptibility	
							*Longusvirus carli*	*Molestusvirus kimi*
LR55; *Shingomonas* sp. (isolation host)	Taastrup/ 2021	L9	65.3	4.07	JASPFO000000000	This study	+	+
LR59; *Shingomonas* sp.	Taastrup/ 2021	L9	65.3	4.2	JASPFL000000000	This study	-	(+) ^d^
LR60; *Shingomonas* sp.	Taastrup/ 2021	L9	66.6	3.9	JASPFK000000000	This study	-	-
LR61; *Shingomonas* sp.	Taastrup/ 2021	L9	65.2	4.4	JASPFJ000000000	This study	-	-
LR57; *Sphingomonas aurantiaca*	Taastrup/ 2021	L9	66.1	4.2	JASPFN000000000	This study	-	-
LR58; *Sphingomonas aerolata*	Taastrup/ 2021	L9	66.1	4.3	JASPFM000000000	This study	-	-
22S1L9-1; *Sphingomonas aerolata*	Soenderborg/ 2022	L9	66.3	4.3	JASPFQ000000000	This study	-	+
22R1PE-11; *Sphingomonas aurantiaca*	Ringsted/2022	PE	66.2	4.3	JASPFT000000000	This study	-	-
22L1PE-1; *Sphingomonas aerolata*	Loekken/2022	PE	66.2	4.2	CP128316	This study	-	-
22R3R2A-7; *Sphingomonas* sp.	Randers/2022	R2A	66.3	4.1	JASPFR000000000	This study	-	-
22R1R2A-14; *Sphingomonas aerolata*	Randers/2022	R2A	66.1	4.2	JASPFS000000000	This study	-	-
22L2VL55-5; *Sphingomonas aerolata*	Loekken/2022	VL55	66.0	4.1	JASPFU000000000	This study	-	(+) ^d^
22L2VL55-3; *Sphingomonas* sp.	Loekken/2022	VL55	65.0	4.1	JASPFV000000000	This study	-	-
22S1VL55-1; *Sphingomonas aurantiaca*	Soenderborg/ 2022	VL55	66.0	4.3	JASPFP000000000	This study	-	-
*Sphingomonas* sp. PM2	-	-	-	-	-	[76]	-	-
*Shingomonas herbicidovorans* MH	-	-	-	-	-	[77]	-	-
*Shingomonas wittichii* RW1	-	-	-	-	-	[59]	-	-

^a^ Coordinates for Danish wheat field locations are as follows: Taastrup (55°40′12.2″ N 12°18′13.0″ E); Ringsted (55°24′00.5″ N 11°49′29.9″ E); Loekken (57°23′38.3″ N 9°51′06.0″ E); Soenderborg (54°58′04.1″ N 9°42′22.6″ E); Randers (56°21′58.3″ N 10°06′49.5″ E). ^b^ Abbreviations for initial isolation medium are as follows: L9—L9 minimal medium [34]; PE—Plant Extract medium (this study); R2A—R2A minimal medium [35]; VL55—VL55 medium [48]. Isolates were obtained from either the Sheriff, Rembrandt, Kvium, or Heerup winter wheat cultivars. ^c^ Assembled and annotated genomes were submitted to NCBI GenBank [39] under the Bioproject PRJNA976731. ^d^ Clearing zones on lawns of these *Sphingomonas* isolates were observed at concentrations of ~10^8^ PFUs/mL and above, but formation of individual plaques was not observed.

Surprisingly, the phage *Molestusvirus kimi* formed clear, individual plaques at concentrations of ~10 PFUs/mL and above when tested on one of the *Sphingomonas aerolota* wheat phyllosphere isolates (strain no. 22S1L9-1; Table 1) sampled from a different location and year compared to that of the original host. The EOP for this isolate was 1.9 ± 0.2, indicating a slightly better infection potential of the phage *Molestusvirus kimi* on this *Sphingomonas* isolate compared to the original host. 

In addition, the phage *Molestusvirus kimi* was able to generate clear zones of phage lytic activity at concentrations above 10^8^ PFUs/mL on two of the tested wheat phyllosphere *Sphingomonas* isolates (Table 1), suggesting lysis from without or abortive infection [78]. 

Both phages were tested against an additional 15 *Sphingomonas aerolata* strains, isolated from different wheat trial fields; however, none showed susceptibility to either of the phages, which further confirms the complexity of phage–host interactions as well as the specificity and narrow host range of these phages.

These observations resemble previous studies where narrow host ranges were reported for phages infecting bacterial members of both the *Sphingomonas* genus [25] and the *Sphingobium* genus [29]. Typically, most phages targeting other types of environmental bacteria are found to be host specific without the ability to infect a wider range of bacteria across species and genus, i.e., to trespass generic boundaries [73,79,80,81]. 

## 4. Conclusions

Bacterial members of the *Sphingomonas* genus hold immense environmental significance and have garnered considerable attention as candidates for a wide range of biotechnological applications. These bacteria are widely distributed across diverse natural environments, playing a vital role as keystone members of plant phyllosphere microbiomes. Exploring and understanding the intricate interactions between bacteriophages and *Sphingomonas* species not only enhances our fundamental comprehension of natural microbial systems but also unveils promising avenues for future microbiome manipulation strategies.

Currently, the repertoire of phages specifically targeting bacterial members of the *Sphingomonas* genus remains relatively limited. However, in this study, we present the isolation and characterization of three novel phages—*Longusvirus carli*, *Vexovirus birtae*, and *Molestusvirus kimi*—which, for the first time, are shown to target and regulate a *Sphingomonas* member found within the wheat phyllosphere microbiome. 

Phenotypically, two of the phages were found to display a non-prolate (B1) Siphovirus morphotype, which is common among phages isolated from various environmental habitats but less widespread among phages infecting members of the *Sphingomonas* genus. In addition, the isolated phages had a narrow host range like several other phages, and, during infection, both phages displayed long latency periods, which is not uncommon for slow-growing bacterial host strains like the *Sphingomonas* host isolated in this study. 

What is particularly intriguing is that the genotypic analyses of these phages revealed distinct features that distinguish them significantly from known phages cataloged in publicly available databases. Together with other recently reported findings of novel phages isolated from natural environments, these findings not only underscore the tremendous genetic diversity among ecological phages but also highlight the fact that our current understanding barely scratches the surface of this vast landscape.

Hence, the description of these phages represents a valuable addition to the growing database of environmental phage genomes and contributes to the exciting expansion of our taxonomic knowledge concerning bacterial viruses. 

## Data Availability

Publicly available datasets were analyzed in this study. All of the genome sequence data obtained by nanopore sequencing of the *Sphingomonas* strains isolated from the Danish wheat phyllosphere have been deposited in the NCBI GenBank under the BioProject ID PRJNA976731. Accession numbers of individual isolates are listed in Table 1. Assembled and annotated genomes of phages *Longusvirus carli*, *Vexovirus birtae*, and *Molestusvirus kimi* have been assigned to GenBank with accession numbers OR225223, OR225224, and OR225222, respectively.

## References

[1] Mann N.H. (2005). The third age of phage. PLoS Biol..

[2] Hendrix R.W. (2003). Bacteriophage genomics. Curr. Opin. Microbiol..

[3] Hanlon G.W. (2007). Bacteriophages: An appraisal of their role in the treatment of bacterial infections. Int. J. Antimicrob. Agents.

[4] Batinovic S., Wassef F., Knowler S.A., Rice D.T.F., Stanton C.R., Rose J., Tucci J., Nittami T., Vinh A., Drummond G.R. (2019). Bacteriophages in Natural and Artificial Environments. Pathogens.

[5] Jardillier L., Bettarel Y., Richardot M., Bardot C., Amblard C., Sime-Ngando T., Debroas D. (2005). Effects of viruses and predators on prokaryotic community composition. Microb. Ecol..

[6] Rogovski P., Cadamuro R.D., da Silva R., de Souza E.B., Bonatto C., Viancelli A., Michelon W., Elmahdy E.M., Treichel H., Rodriguez-Lazaro D. (2021). Uses of Bacteriophages as Bacterial Control Tools and Environmental Safety Indicators. Front. Microbiol..

[7] Coffey B., Mills S., Coffey A., McAuliffe O., Ross R.P. (2010). Phage and their lysins as biocontrol agents for food safety applications. Annu. Rev. Food Sci. Technol..

[8] Keen E.C. (2015). A century of phage research: Bacteriophages and the shaping of modern biology. Bioessays.

[9] Stolz A. (2009). Molecular characteristics of xenobiotic-degrading sphingomonads. Appl. Microbiol. Biotechnol..

[10] Asaf S., Numan M., Khan A.L., Al-Harrasi A. (2020). *Sphingomonas*: From diversity and genomics to functional role in environmental remediation and plant growth. Crit. Rev. Biotechnol..

[11] Kim H., Nishiyama W., Kunito T., Senoo K., Kawahara K., Murakami K., Oyaizu H. (1998). High population of *Sphingomonas* species on plant surface. J. Appl. Microbiol..

[12] Yun N.R., Shin Y.K., Hwang S.Y., Kuraishi H., Sugiyama J., Kawahara K. (2000). Chemotaxonomic and phylogenetic analyses of *Sphingomonas* strains isolated from ears of plants in the family Gramineae and a proposal of *Sphingomonas roseoflava* sp. nov. J. Gen. Appl. Microbiol..

[13] Enya J., Shinohara H., Yoshida S., Tsukiboshi T., Negishi H., Suyama K., Tsushima S. (2007). Culturable leaf-associated bacteria on tomato plants and their potential as biological control agents. Microb. Ecol..

[14] Khan A.L., Waqas M., Kang S.M., Al-Harrasi A., Hussain J., Al-Rawahi A., Al-Khiziri S., Ullah I., Ali L., Jung H.Y. (2014). Bacterial endophyte *Sphingomonas* sp. LK11 produces gibberellins and IAA and promotes tomato plant growth. J. Microbiol..

[15] Luo Y., Wang F., Huang Y., Zhou M., Gao J., Yan T., Sheng H., An L. (2019). *Sphingomonas* sp. Cra20 Increases Plant Growth Rate and Alters Rhizosphere Microbial Community Structure of *Arabidopsis thaliana* under Drought Stress. Front. Microbiol..

[16] Asaf S., Khan A.L., Khan M.A., Imran Q.M., Yun B.W., Lee I.J. (2017). Osmoprotective functions conferred to soybean plants via inoculation with *Sphingomonas* sp. LK11 and exogenous trehalose. Microbiol. Res..

[17] Halo B.A., Khan A.L., Waqas M., Al-Harrasi A., Hussain J., Ali L., Adnan M., Lee I.J. (2015). Endophytic bacteria (*Sphingomonas* sp. LK11) and gibberellin can improve *Solanum lycopersicum* growth and oxidative stress under salinity. J. Plant Interact..

[18] Yu F.B., Shan S.D., Luo L.P., Guan L.B., Qin H. (2013). Isolation and characterization of a *Sphingomonas* sp. strain F-7 degrading fenvalerate and its use in bioremediation of contaminated soil. J. Environ. Sci. Health B.

[19] Chen J., Wong M.H., Wong Y.S., Tam N.F. (2008). Multi-factors on biodegradation kinetics of polycyclic aromatic hydrocarbons (PAHs) by *Sphingomonas* sp. a bacterial strain isolated from mangrove sediment. Mar. Pollut. Bull..

[20] Leys N.M., Ryngaert A., Bastiaens L., Verstraete W., Top E.M., Springael D. (2004). Occurrence and phylogenetic diversity of *Sphingomonas* strains in soils contaminated with polycyclic aromatic hydrocarbons. Appl. Environ. Microbiol..

[21] Willison J.C. (2004). Isolation and characterization of a novel sphingomonad capable of growth with chrysene as sole carbon and energy source. FEMS Microbiol. Lett..

[22] Huang H., Lin J., Wang W., Li S. (2022). Biopolymers Produced by *Sphingomonas* Strains and Their Potential Applications in Petroleum Production. Polymers.

[23] Aylward F.O., McDonald B.R., Adams S.M., Valenzuela A., Schmidt R.A., Goodwin L.A., Woyke T., Currie C.R., Suen G., Poulsen M. (2013). Comparison of 26 sphingomonad genomes reveals diverse environmental adaptations and biodegradative capabilities. Appl. Environ. Microbiol..

[24] Santillan J.Y., Rojas N.L., Ghiringhelli P.D., Nobile M.L., Lewkowicz E.S., Iribarren A.M. (2020). Organophosphorus compounds biodegradation by novel bacterial isolates and their potential application in bioremediation of contaminated water. Bioresour. Technol..

[25] Marmo P., Thaller M.C., Di Lallo G., Henrici De Angelis L., Poerio N., De Santis F., Fraziano M., Migliore L., D’Andrea M.M. (2020). Characterization of vB_StuS_MMDA13, a Newly Discovered Bacteriophage Infecting the Agar-Degrading Species *Sphingomonas turrisvirgatae*. Viruses.

[26] Jiang S.C., Kellogg C.A., Paul J.H. (1998). Characterization of marine temperate phage-host systems isolated from Mamala Bay, Oahu, Hawaii. Appl. Environ. Microbiol..

[27] Wolf A., Wiese J., Jost G., Witzel K.P. (2003). Wide geographic distribution of bacteriophages that lyse the same indigenous freshwater isolate (*Sphingomonas* sp. strain B18). Appl. Environ. Microbiol..

[28] Nakayama N., Tsuge T., Asakawa S., Kimura M. (2009). Morphology, host range and phylogenetic diversity of *Sphingomonas* phages in the floodwater of a Japanese paddy field. Soil Sci. Plant Nutr..

[29] Nielsen T.K., Carstens A.B., Browne P., Lametsch R., Neve H., Kot W., Hansen L.H. (2017). The first characterized phage against a member of the ecologically important sphingomonads reveals high dissimilarity against all other known phages. Sci. Rep..

[30] Curtis T., Halford N.G. (2014). Food security: The challenge of increasing wheat yield and the importance of not compromising food safety. Ann. Appl. Biol..

[31] Sohrabi R., Paasch B.C., Liber J.A., He S.Y. (2023). Phyllosphere Microbiome. Annu. Rev. Plant Biol..

[32] Carlstrom C.I., Field C.M., Bortfeld-Miller M., Muller B., Sunagawa S., Vorholt J.A. (2019). Synthetic microbiota reveal priority effects and keystone strains in the Arabidopsis phyllosphere. Nat. Ecol. Evol..

[33] Hawkes C.V., Kjoller R., Raaijmakers J.M., Riber L., Christensen S., Rasmussen S., Christensen J.H., Dahl A.B., Westergaard J.C., Nielsen M. (2021). Extension of Plant Phenotypes by the Foliar Microbiome. Annu. Rev. Plant Biol..

[34] Yim M.S., Yau Y.C., Matlow A., So J.S., Zou J., Flemming C.A., Schraft H., Leung K.T. (2010). A novel selective growth medium-PCR assay to isolate and detect *Sphingomonas* in environmental samples. J. Microbiol. Methods.

[35] Reasoner D.J., Geldreich E.E. (1985). A new medium for the enumeration and subculture of bacteria from potable water. Appl. Environ. Microbiol..

[36] Kolmogorov M., Yuan J., Lin Y., Pevzner P.A. (2019). Assembly of long, error-prone reads using repeat graphs. Nat. Biotechnol..

[37] Seemann T. (2014). Prokka: Rapid prokaryotic genome annotation. Bioinformatics.

[38] Kanehisa M., Sato Y., Morishima K. (2016). BlastKOALA and GhostKOALA: KEGG Tools for Functional Characterization of Genome and Metagenome Sequences. J. Mol. Biol..

[39] Benson D.A., Karsch-Mizrachi I., Lipman D.J., Ostell J., Sayers E.W. (2011). GenBank. Nucleic Acids Res..

[40] Van Twest R., Kropinski A.M. (2009). Bacteriophage enrichment from water and soil. Methods Mol. Biol..

[41] Kropinski A.M., Mazzocco A., Waddell T.E., Lingohr E., Johnson R.P. (2009). Enumeration of bacteriophages by double agar overlay plaque assay. Methods Mol. Biol..

[42] Carstens A.B., Djurhuus A.M., Kot W., Hansen L.H. (2019). A novel six-phage cocktail reduces Pectobacterium atrosepticum soft rot infection in potato tubers under simulated storage conditions. FEMS Microbiol. Lett..

[43] Bonilla N., Rojas M.I., Netto Flores Cruz G., Hung S.H., Rohwer F., Barr J.J. (2016). Phage on tap-a quick and efficient protocol for the preparation of bacteriophage laboratory stocks. PeerJ.

[44] Olsen N.S., Lametsch R., Wagner N., Hansen L.H., Kot W. (2022). Salmonella phage akira, infecting selected Salmonella enterica Enteritidis and Typhimurium strains, represents a new lineage of bacteriophages. Arch. Virol..

[45] Ostergaard Breum S., Neve H., Heller K.J., Vogensen F.K. (2007). Temperate phages TP901-1 and phiLC3, belonging to the P335 species, apparently use different pathways for DNA injection in Lactococcus lactis subsp. cremoris 3107. FEMS Microbiol. Lett..

[46] Kropinski A.M. (2018). Practical Advice on the One-Step Growth Curve. Methods Mol. Biol..

[47] Hyman P., Abedon S.T. (2009). Practical methods for determining phage growth parameters. Methods Mol. Biol..

[48] Sait M., Hugenholtz P., Janssen P.H. (2002). Cultivation of globally distributed soil bacteria from phylogenetic lineages previously only detected in cultivation-independent surveys. Environ. Microbiol..

[49] Kutter E. (2009). Phage host range and efficiency of plating. Methods Mol. Biol..

[50] Aziz R.K., Bartels D., Best A.A., DeJongh M., Disz T., Edwards R.A., Formsma K., Gerdes S., Glass E.M., Kubal M. (2008). The RAST Server: Rapid annotations using subsystems technology. BMC Genom..

[51] Moraru C., Varsani A., Kropinski A.M. (2020). VIRIDIC-A Novel Tool to Calculate the Intergenomic Similarities of Prokaryote-Infecting Viruses. Viruses.

[52] Altschul S.F., Gish W., Miller W., Myers E.W., Lipman D.J. (1990). Basic local alignment search tool. J. Mol. Biol..

[53] Altschul S.F., Madden T.L., Schaffer A.A., Zhang J., Zhang Z., Miller W., Lipman D.J. (1997). Gapped BLAST and PSI-BLAST: A new generation of protein database search programs. Nucleic Acids Res..

[54] Gilchrist C.L.M., Chooi Y.H. (2021). Clinker & clustermap.js: Automatic generation of gene cluster comparison figures. Bioinformatics.

[55] Chaumeil P.A., Mussig A.J., Hugenholtz P., Parks D.H. (2019). GTDB-Tk: A toolkit to classify genomes with the Genome Taxonomy Database. Bioinformatics.

[56] Arndt D., Grant J.R., Marcu A., Sajed T., Pon A., Liang Y., Wishart D.S. (2016). PHASTER: A better, faster version of the PHAST phage search tool. Nucleic Acids Res..

[57] Bertazzi P.A., Consonni D., Bachetti S., Rubagotti M., Baccarelli A., Zocchetti C., Pesatori A.C. (2001). Health effects of dioxin exposure: A 20-year mortality study. Am. J. Epidemiol..

[58] Colquhoun D.R., Hartmann E.M., Halden R.U. (2012). Proteomic profiling of the dioxin-degrading bacterium *Sphingomonas wittichii* RW1. J. Biomed. Biotechnol..

[59] Wittich R.M., Wilkes H., Sinnwell V., Francke W., Fortnagel P. (1992). Metabolism of dibenzo-p-dioxin by *Sphingomonas* sp. strain RW1. Appl. Environ. Microbiol..

[60] Rocha E.P., Danchin A. (2002). Base composition bias might result from competition for metabolic resources. Trends Genet..

[61] Carstens A.B., Kot W., Lametsch R., Neve H., Hansen L.H. (2016). Characterisation of a novel enterobacteria phage, CAjan, isolated from rat faeces. Arch. Virol..

[62] Thiaville J.J., Kellner S.M., Yuan Y., Hutinet G., Thiaville P.C., Jumpathong W., Mohapatra S., Brochier-Armanet C., Letarov A.V., Hillebrand R. (2016). Novel genomic island modifies DNA with 7-deazaguanine derivatives. Proc. Natl. Acad. Sci. USA.

[63] Hutinet G., Kot W., Cui L., Hillebrand R., Balamkundu S., Gnanakalai S., Neelakandan R., Carstens A.B., Fa Lui C., Tremblay D. (2019). 7-Deazaguanine modifications protect phage DNA from host restriction systems. Nat. Commun..

[64] Kot W., Olsen N.S., Nielsen T.K., Hutinet G., de Crecy-Lagard V., Cui L., Dedon P.C., Carstens A.B., Moineau S., Swairjo M.A. (2020). Detection of preQ0 deazaguanine modifications in bacteriophage CAjan DNA using Nanopore sequencing reveals same hypermodification at two distinct DNA motifs. Nucleic Acids Res..

[65] Turner D., Shkoporov A.N., Lood C., Millard A.D., Dutilh B.E., Alfenas-Zerbini P., van Zyl L.J., Aziz R.K., Oksanen H.M., Poranen M.M. (2023). Abolishment of morphology-based taxa and change to binomial species names: 2022 taxonomy update of the ICTV bacterial viruses subcommittee. Arch. Virol..

[66] Jurczak-Kurek A., Gasior T., Nejman-Falenczyk B., Bloch S., Dydecka A., Topka G., Necel A., Jakubowska-Deredas M., Narajczyk M., Richert M. (2016). Biodiversity of bacteriophages: Morphological and biological properties of a large group of phages isolated from urban sewage. Sci. Rep..

[67] Demuth J., Neve H., Witzel K.P. (1993). Direct electron microscopy study on the morphological diversity of bacteriophage populations in lake plusssee. Appl. Environ. Microbiol..

[68] Ackermann H.W., Eisenstark A. (1974). The present state of phage taxonomy. Intervirology.

[69] Denes T., Vongkamjan K., Ackermann H.W., Moreno Switt A.I., Wiedmann M., den Bakker H.C. (2014). Comparative genomic and morphological analyses of Listeria phages isolated from farm environments. Appl. Environ. Microbiol..

[70] Sepulveda-Robles O., Kameyama L., Guarneros G. (2012). High diversity and novel species of *Pseudomonas aeruginosa* bacteriophages. Appl. Environ. Microbiol..

[71] Lu L., Cai L., Jiao N., Zhang R. (2017). Isolation and characterization of the first phage infecting ecologically important marine bacteria Erythrobacter. Virol. J..

[72] Yang Y., Cai L., Ma R., Xu Y., Tong Y., Huang Y., Jiao N., Zhang R. (2017). A Novel Roseosiphophage Isolated from the Oligotrophic South China Sea. Viruses.

[73] Weinbauer M.G. (2004). Ecology of prokaryotic viruses. FEMS Microbiol. Rev..

[74] Li B., Zhang S., Long L., Huang S. (2016). Characterization and Complete Genome Sequences of Three N4-Like Roseobacter Phages Isolated from the South China Sea. Curr. Microbiol..

[75] Abedon S.T. (2023). Bacteriophage Adsorption: Likelihood of Virion Encounter with Bacteria and Other Factors Affecting Rates. Antibiotics.

[76] Qiu S.R., Gozdereliler E., Weyrauch P., Lopez E.C.M., Kohler H.P.E., Sorensen S.R., Meckenstock R.U., Elsner M. (2014). Small C-13/C-12 Fractionation Contrasts with Large Enantiomer Fractionation in Aerobic Biodegradation of Phenoxy Acids. Environ. Sci. Technol..

[77] Kohler H.P. (1999). *Sphingomonas herbicidovorans* MH: A versatile phenoxyalkanoic acid herbicide degrader. J. Ind. Microbiol. Biotechnol..

[78] Abedon S.T. (2011). Lysis from without. Bacteriophage.

[79] Koskella B., Meaden S. (2013). Understanding bacteriophage specificity in natural microbial communities. Viruses.

[80] Flores C.O., Meyer J.R., Valverde S., Farr L., Weitz J.S. (2011). Statistical structure of host-phage interactions. Proc. Natl. Acad. Sci. USA.

[81] Poullain V., Gandon S., Brockhurst M.A., Buckling A., Hochberg M.E. (2008). The evolution of specificity in evolving and coevolving antagonistic interactions between a bacteria and its phage. Evolution.

